# Supplementary material for: Efficient protein production inspired by how spiders make silk
Source: Nat Commun. 2017 May 23;8:15504. doi: 10.1038/ncomms15504 (PMC5457526; doi:10.1038/ncomms15504)
Supplement: Supplementary Information — Supplementary Figures, Supplementary Tables and Supplementary References [file ncomms15504-s1.pdf]

SUPPLEMENTARY FIGURES

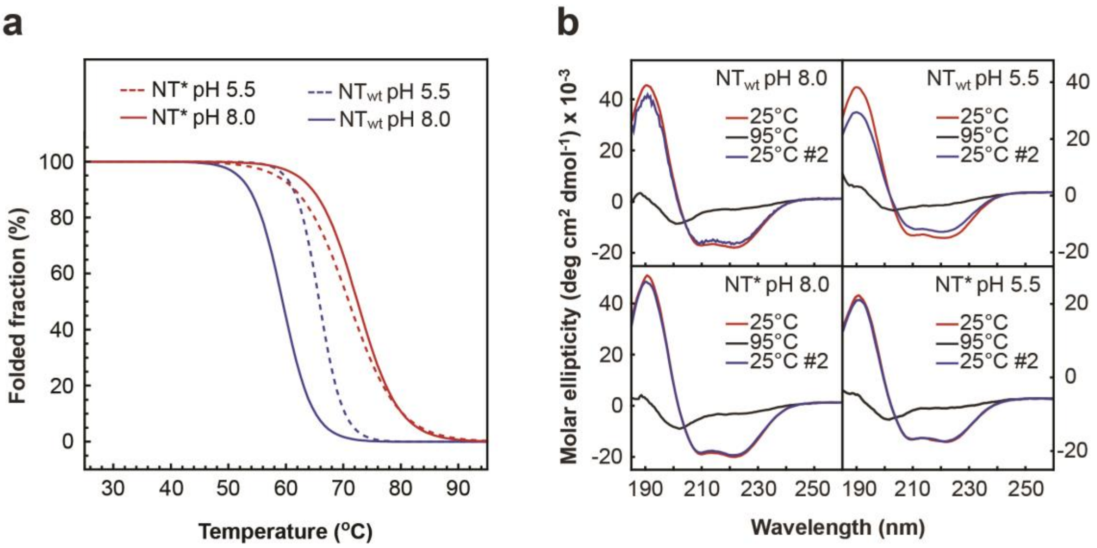

Supplementary Figure 1: CD spectroscopy measurement of stability and refolding capacity. (a) The CD signal at 222 nm was converted to fraction folded, as described in the Methods section, plotted as a function of temperature (^oC) and fitted to a two-state unfolding model to obtain the melting temperatures (T_m) at the equilibration points. The obtained T_m values were 59.4 ^oC or 65.8 ^oC for NT_{wt} at pH 8.0 (blue solid line) or pH 5.5 (blue dotted line), respectively, and 72.4 ^oC or 70.9 ^oC for NT* at pH 8.0 (red solid line) or pH 5.5 (red dotted line), respectively. **(b)** The molar ellipticity was scanned from 185 nm to 260 nm at 25 °C (red), 95 °C (black), and again at 25 °C after cooling (blue) at pH 8.0 and pH 5.5 for NT_{wt} (upper panels) and NT* (lower panels). The graphs represent smoothed averages of four measurements.

NT_{wt}

SHTTPWTNPGLAENFMNSFMQGLSSMPGFTASQLDDMSTIAQSMVQSIQSLAAQGRTSPNKLQALN
MAFASSMAEIAASEEGGGSLSTKTSSIASAMSNAFLQTTGVVNQPFINEITQLVSMFAQAGMNDVSA

NT*

SHTTPWTNPGLAENFMNSFMQGLSSMPGFTASQLDKMSTIAQSMVQSIQSLAAQGRTSPNDLQALN
MAFASSMAEIAASEEGGGSLSTKTSSIASAMSNAFLQTTGVVNQPFINEITQLVSMFAQAGMNDVSA

NT*-rSP-C33Leu

MGHHHHHHMSHTTPWTNPGLAENFMNSFMQGLSSMPGFTASQLDKMSTIAQSMVQSIQSLAAQGR
TSPNDLQALNMAFASSMAEIAASEEGGGSLSTKTSSIASAMSNAFLQTTGVVNQPFINEITQLVSMFAQ
AGMNDVSAGNSMIPSSPVHLKRLKLLLLLLLLILLLILGALLLGL

NT*-rKL4

MGHHHHHHMSHTTPWTNPGLAENFMNSFMQGLSSMPGFTASQLDKMSTIAQSMVQSIQSLAAQGR
TSPNDLQALNMAFASSMAEIAASEEGGGSLSTKTSSIASAMSNAFLQTTGVVNQPFINEITQLVSMFAQ
AGMNDVSAGNSMKLLLLKLLLLKLLLLKLLLLK

NT*-rfhSP-D

MGHHHHHHMSHTTPWTNPGLAENFMNSFMQGLSSMPGFTASQLDKMSTIAQSMVQSIQSLAAQGR
TSPNDLQALNMAFASSMAEIAASEEGGGSLSTKTSSIASAMSNAFLQTTGVVNQPFINEITQLVSMFAQ
AGMNDVSAGNSLEALFQGPGIPGECGEKGEPGERGPPGLPAHLDEELQATLHDFRHQILQTRGALSL
QGSIMTVGEKVFSSNGQSITFDAIQEACARAGGRIAVPRNPEENEAIASFVKKYNTYAYVGLTEGPSPG
DFRYSDGTPVNYTNWYRGEPAGRGKEQCVEMYTDGQWNDRNCLYSRLTICEF

NT*-rCCK-58

MGHHHHHHMSHTTPWTNPGLAENFMNSFMQGLSSMPGFTASQLDKMSTIAQSMVQSIQSLAAQGR
TSPNDLQALNMAFASSMAEIAASEEGGGSLSTKTSSIASAMSNAFLQTTGVVNQPFINEITQLVSMFAQ
AGMNDVSAGNSLEALFQGVSQRTDGESRAHLGALLARYIQQARKAPSGRMSIVKNLQNLDPSHRISD
RDYMGWMDF

NT*-rAβ1-42

MGHHHHHHMSHTTPWTNPGLAENFMNSFMQGLSSMPGFTASQLDKMSTIAQSMVQSIQSLAAQGR
TSPNDLQALNMAFASSMAEIAASEEGGGSLSTKTSSIASAMSNAFLQTTGVVNQPFINEITQLVSMFAQ
AGMNDVSAGNSENLYFQDAEFRHDSGYEVHHQKLVFFAEDVGSNKGAIIGLMVGGVVIA

NT*-rhCAP-18

MGHHHHHHMSHTTPWTNPGLAENFMNSFMQGLSSMPGFTASQLDKMSTIAQSMVQSIQSLAAQGR
TSPNDLQALNMAFASSMAEIAASEEGGGSLSTKTSSIASAMSNAFLQTTGVVNQPFINEITQLVSMFAQ
AGMNDVSAGNSLVPRGSQVLSYKEAVLRAIDGINQRSSDANLYRLLDLDPRPTMDGDPDTPKPVSFT
VKETVCPRTTQQSPEDCDFKKDGLVKRCMGTVTLNQARGSFDISCDKDNKRFALLGDFFRKSKEKIG
KEFKRIVQRIKDFLRNLVPRTES

NT*-c-Myc-rβ17

MGHHHHHHMSHTTPWTNPGLAENFMNSFMQGLSSMPGFTASQLDKMSTIAQSMVQSIQSLAAQGR
TSPNDLQALNMAFASSMAEIAASEEGGGSLSTKTSSIASAMSNAFLQTTGVVNQPFINEITQLVSMFAQ
AGMNDVSAGNSLVPRGSMGEQKLISEEDLGMQISMDYEIKFHGDGDNFDLNLDDSGGDLQLQIRGP
GGRVHVHIHSSSGKVDFHVNNDGGDVEVKMH

NT*-rSP-C_{ss}

MGHHHHHHMSHTTPWTNPGLAENFMNSFMQGLSSMPGFTASQLDKMSTIAQSMVQSIQSLAAQGR
TSPNDLQALNMAFASSMAEIAASEEGGGSLSTKTSSIASAMSNAFLQTTGVVNQPFINEITQLVSMFAQ
AGMNDVSAGNSMFGIPSSPVHLKRLLIVVVVVVLIVVVIVGALLLGL

Supplementary Figure 2: Amino acid sequences of NT_{wt}, NT* and fusion proteins. The His_6 affinity purification tag is shown in orange, NT is shown in grey, and the fused target peptides or proteins are shown in green. Cleavage sites for CNBr (M), 3C protease (LEALFQG), tobacco etch virus (TEV) protease (ENLYFQ) or thrombin (LVPRGS) are underlined in the fusion proteins. The D40K and K65D mutations are shown in red in the sequence corresponding to NT*.

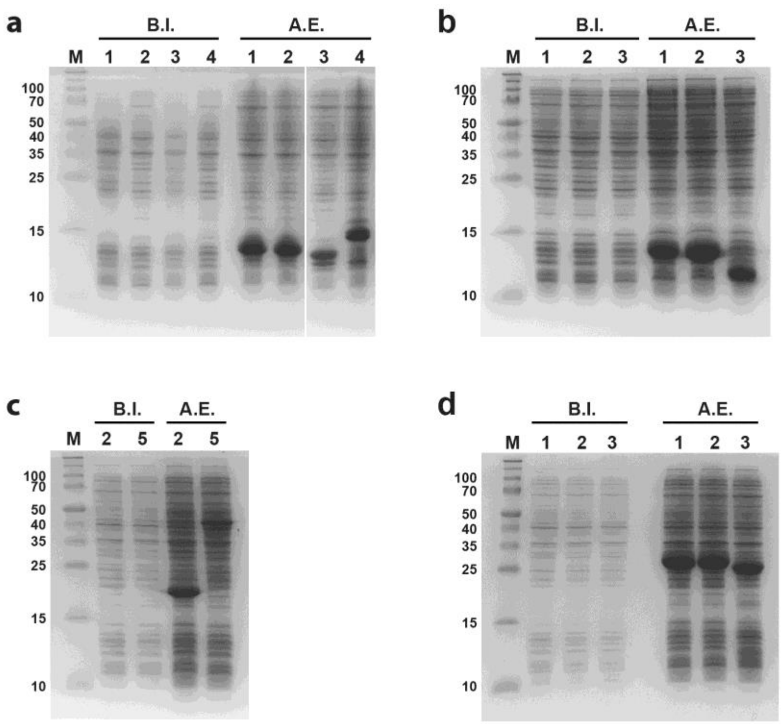

Supplementary Figure 3: Expression analysis of fusion proteins. Target peptides or proteins were fused C-terminally of NT_{wt} (1), NT* (2), PGB1 (3), Trx (4) or MBP (5). SDS-PAGE samples were taken before induction (B.I.) and after over-night expression (A.E.) of **(a)** rSP-C33Leu, **(b)** rKL4, **(c)** rCCK58 and **(d)** rfhSP-D fusion proteins. The molecular weights in kDa of a protein standard (lane M) are given to the left of each gel figure..

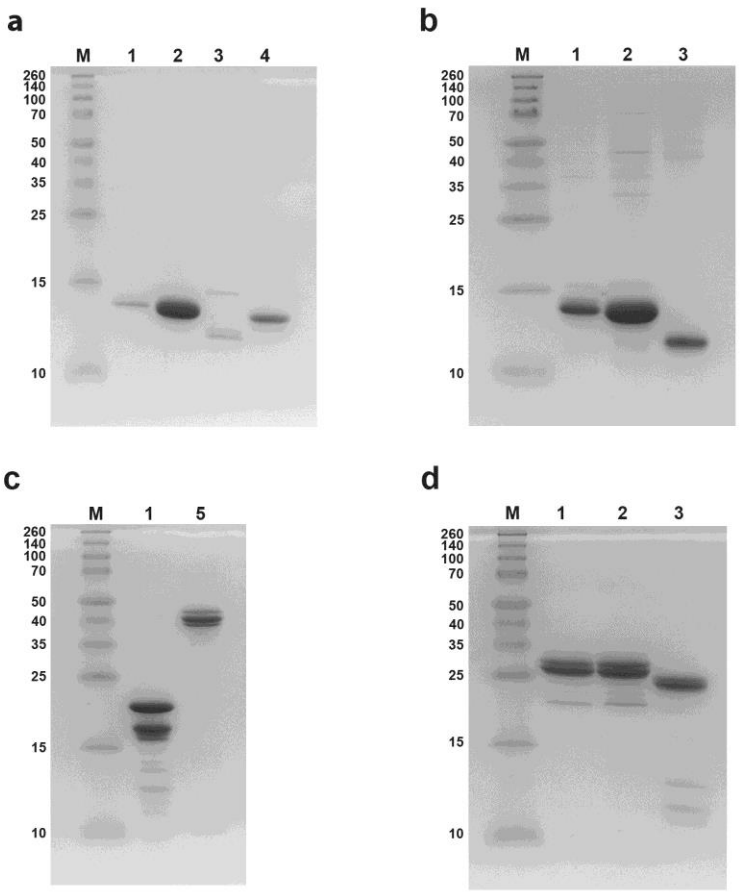

Supplementary Figure 4: Comparative purification of fusion proteins. Target peptides or proteins were fused C-terminally of NT_{wt} (1), NT* (2), PGB1 (3), Trx (4) or MBP (5). Fusion proteins were purified on Ni-sepharose using identical protocols and SDS-PAGE analysis of the purified protein fraction is shown for **(a)** rSP-C33Leu, **(b)** rKL4, **(c)** rCCK58 and **(d)** rfhSP-D fusion proteins. The molecular weights in kDa of a protein standard (lane M) are given to the left of each gel figure.

a
b
c

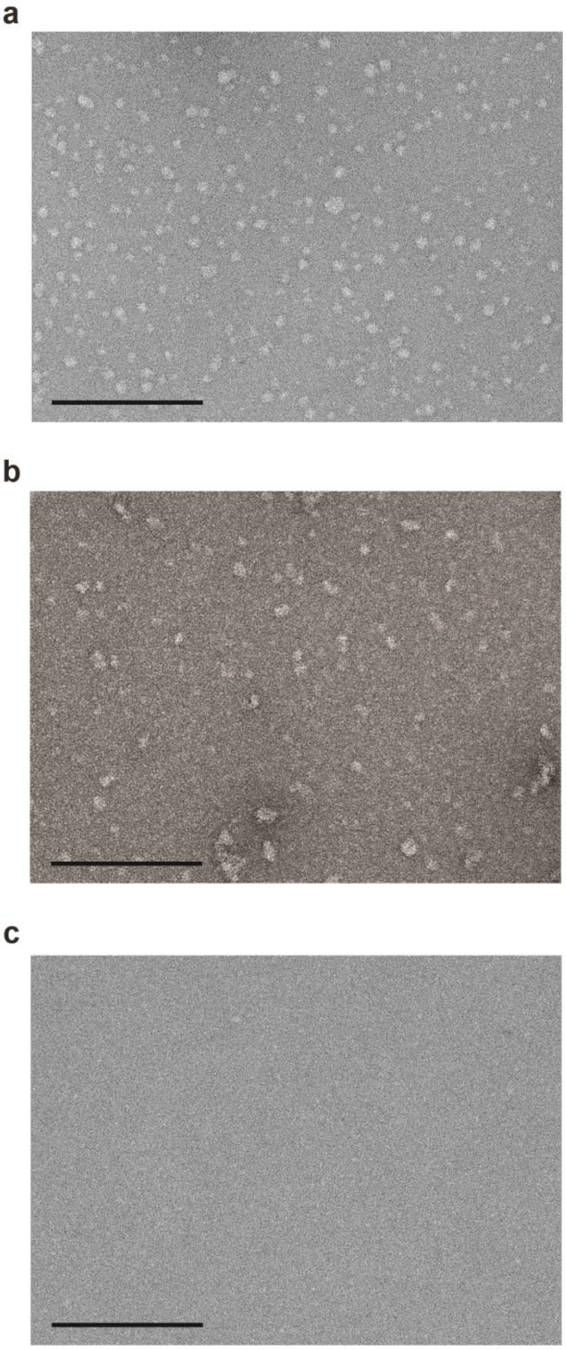

Supplementary Figure 5: NT* in fusion with TM peptides arranges into micelle-like particles. TEM of negatively stained **(a)** NT*-rSP-C33Leu fusion protein and **(b)** NT*-rKL4 fusion protein shows 10-15 nm particles. **(c)** TEM of negatively stained NT* at the same concentration served as negative control. Scale bar = 200 nm.

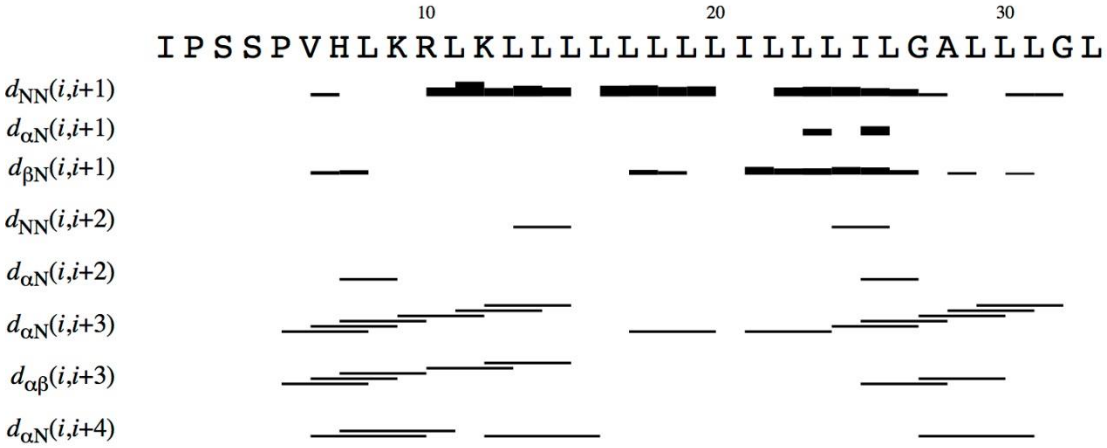

Supplementary Figure 6: Amino acid sequence of rSP-C33Leu together with sequential and medium range NOE connectivities. The first three rows depict the sequential connectivities d_{NN}, $d_{\alpha N}$ and $d_{\beta N}$ where bar height reflects NOE intensity. Medium range connectivities d_{NN}(i, i+2), $d_{\alpha N}$(i, i+3), $d_{\alpha N}$(i, i+4) and $d_{\alpha\beta}$(i, i+3) are represented by lines starting and ending at the positions of the interacting residues.

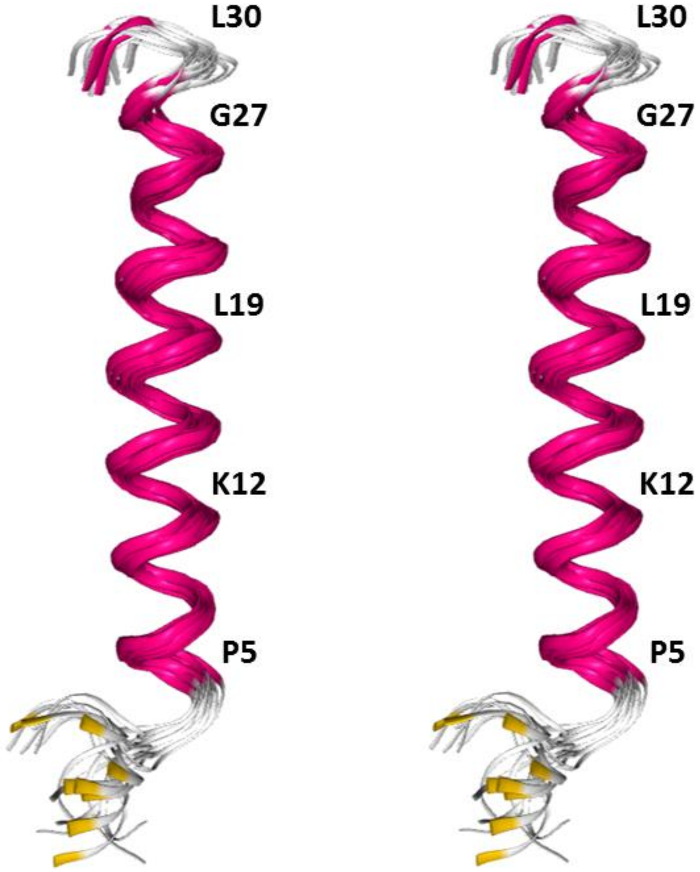

Supplementary Figure 7: Structural characterization of rSP-C33Leu. Stereo view of the 20 best energy-refined conformers representing the three-dimensional structure of rSpC-33Leu in organic solvent.

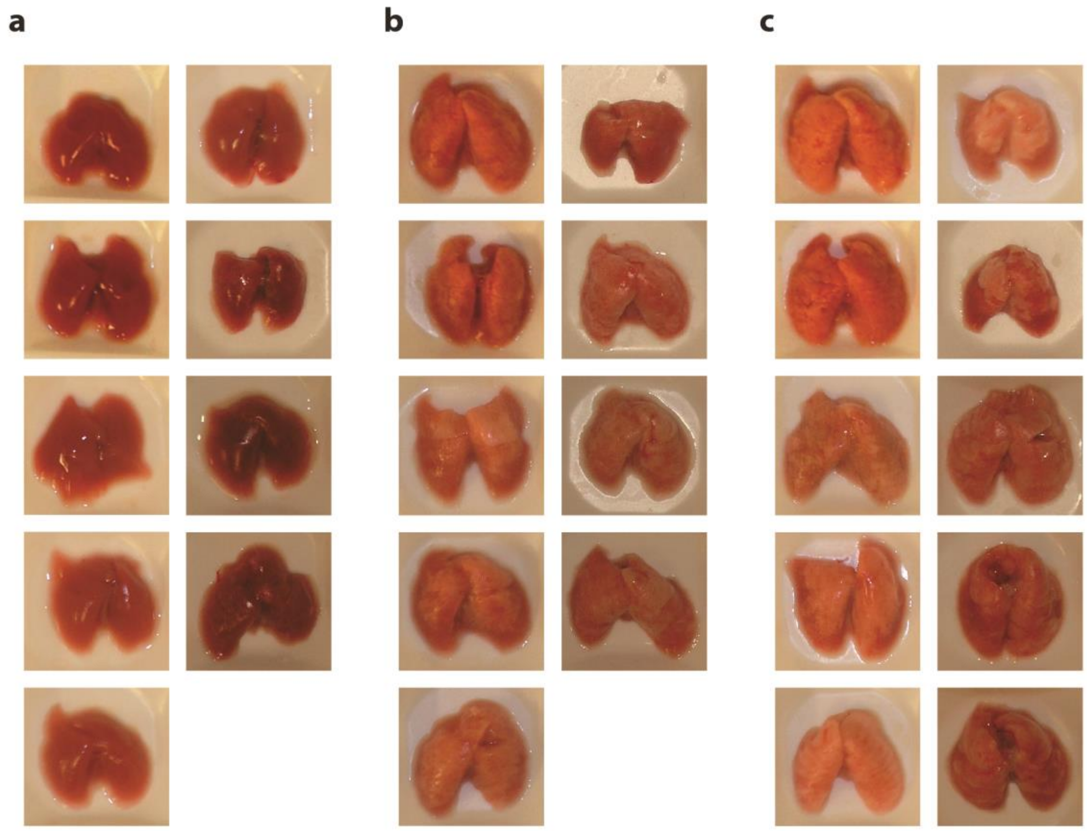

Supplementary Figure 8: Lung appearances after treatment with 2% rSP-C33Leu in DPPC:POPG or Curosurf®. Photographs are shown of whole lungs from all included animals in the **(a)** non-treated control group, **(b)** after treatment with 2% rSP-C33Leu in DPPC:POPG (68:31) or **(c)** treatment with Curosurf®.

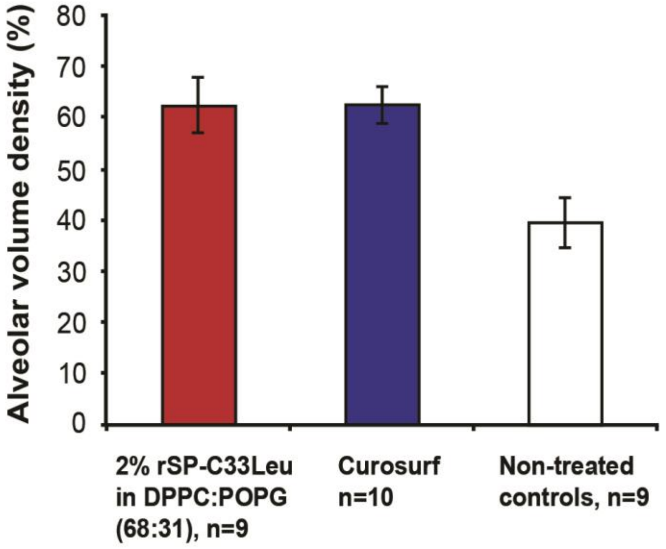

Supplementary Figure 9: Alveolar volume density measured by computer-aided image analysis of excised lungs. Preterm newborn rabbits treated with 2% rSP-C33Leu in DPPC:POPG (68:31) compared to Curosurf® treated or non-treated controls. The bars represent mean values ± s.d. as indicated by error bars and n denotes the number of animals. Treatment with 2% rSP-C33Leu in DPPC:POPG resulted in alveolar volume densities equal to the positive control Curosurf® and both differed significantly from non-treated controls (Newman-Keuls test, $p < 0.0005$).

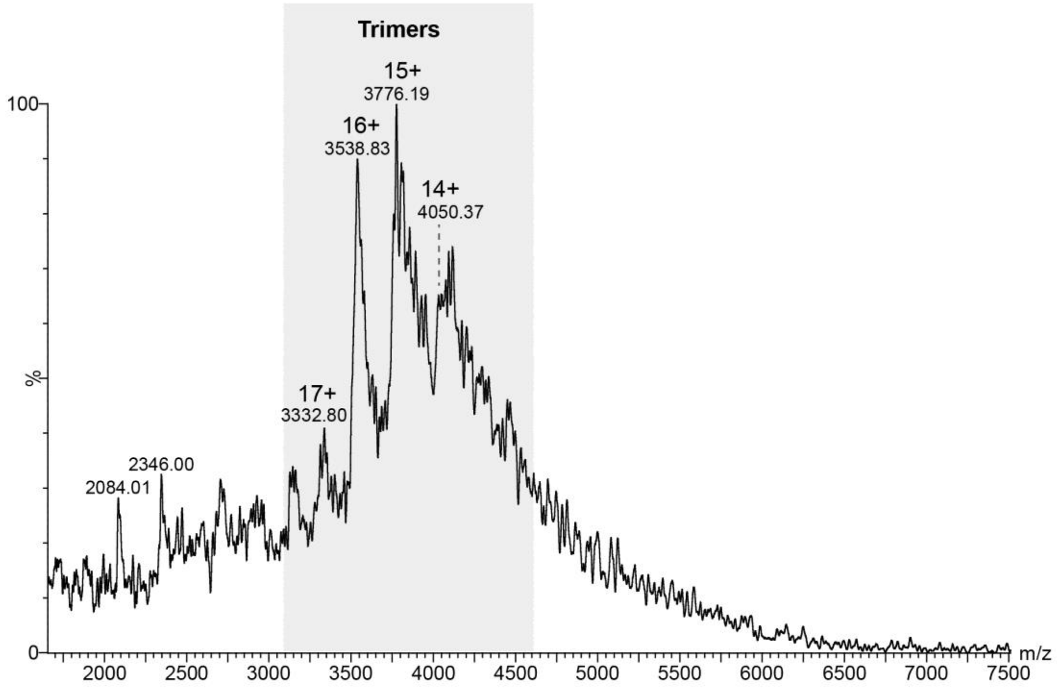

Supplementary Figure 10: ESI-MS spectrum of rfhSP-D. The spectrum was recorded on SEC separated rfhSP-D from the main eluting peak and shows predominantly trimers with 15-16 charges.

SUPPLEMENTARY TABLES

Supplementary Table 1: Protein yields after Ni-Sepharose purification of proteins and peptides in fusion with different solubility tags

Target protein	Fusion partner	Purified fusion protein yield (mg per L culture)[a]	Reference[b]
rSP-C33Leu	NT*	284	
	NT_{wt}	93	
	PGB1	56	
	Trx	43	
rKL4	NT*	428	
	NT_{wt}	212	
	PGB1	56	
$rSP\text{-}C_{ss}$[c]	NT*	59	
	SN	4	Ref[1]
rfhSP-D	NT*	276	
	NT_{wt}	221	
	PGB1	160	
rCCK-58	NT*	142	
	MBP	53	
rhCAP18	NT*	14	
	NT_{wt}	14	
rAβ1-40	NT*	200	
	$(NANP)_{19}$	100	Ref[2]
	IFABP	40	Ref[3]
	GST	95	Ref[4]
rAβ1-42	NT*	174	
	$(NANP)_{19}$	100	Ref[2]
	GST	46	Ref[5]
	GST	9	Ref[6]
	IFABP	40	Ref[3]
	Ub	25	Ref[7]
rβ17	NT*	228	
	PGB1	92	

[a] The yields were calculated for purified fusion protein before removal of the solubility tag
[b] Data without a literature reference were determined in this study
[c] Compared to an SP-C analogue with the two Cys residues exchanged for Phe instead of Ser

Supplementary Table 2: ^{1}H chemical shift values for rSP-C33Leu solubilized in $CDCl_3/CD_3OD$/0.1M HCl 32:64:5 (v/v) at 25 °C

Residue	NH	αH	βH	Others
Ile 1	ni	4.11	ni	γCH_2 1.26; γCH_3 1.12
Pro 2	-	4.50	2.12; 2.32	γCH_2 2.03; δCH_2 3.69; 3.83
Ser 3	8.11	4.47	3.82; 3.93	-
Ser 4	8.42	4.78	3.96; 4.16	-
Pro 5	-	4.36	2.19; 2.43	γCH_2 2.04; 2.09; δCH_2 4.00; 4.06
Val 6	7.71	3.72	2.13	γCH_3 0.98; 1.07
His 7	7.92	4.61	3.44; 3.49	$H\delta_2$ 7.47; $H\varepsilon_1$ 8.84
Leu 8	8.12	4.04	1.78; 1.85	δCH_3 0.98
Lys 9	8.12	3.95	1.98; 2.03	γCH_2 1.68; δCH_2 1.76; εCH_2 2.95; ζNH_2 7.99
Arg 10	8.08	4.05	1.98; 2.04	γCH_2 1.67; δCH_2 3.26; 3.31; εNH_2 7.54
Leu 11	8.02	4.05	ni	δCH_3 0.90
Lys 12	8.15	3.91	1.98, 2.04	γCH_2 1.45 ; δCH_2 1.69; 1.75; εCH_2 2.91; ζNH_2 8.02
Leu 13	7.99	4.11	1.76; 1.87	δCH_3 0.97
Leu 14	8.11	4.09	1.81; 1.91	δCH_3 0.96; 0.98
Leu 15	8.24	4.08	1.92	Hγ 1.66; δCH_3 0.93
Leu 16	8.07	4.10	1.86	δCH_3 0.96
Leu 17	8.24	4.09	1.86	δCH_3 0.97
Leu 18	8.31	4.06	1.93	Hγ 1.74; δCH_3 0.94
Leu 19	8.18	4.08	1.83; 1.88	δCH_3 0.95
Leu 20	8.26	4.06	1.86; 1.93	δCH_3 0.94
Ile 21	8.31	3.66	2.10	γCH_2 1.17; γCH_3 0.97; δCH_3 0.89
Leu 22	8.28	4.06	1.94	δCH_3 0.94
Leu 23	8.44	4.06	1.98; 2.07	Hγ 1.60; δCH_3 0.92
Leu 24	8.36	4.13	1.97; 2.11	Hγ 1.62; δCH_3 0.92
Ile 25	8.42	3.69	2.08	γCH_2 1.11; γCH_3 0.95; δCH_3 0.88
Leu 26	8.64	4.07	1.72; 1.87	δCH_3 0.94
Gly 27	8.56	3.75; 3.83	-	-
Ala 28	8.06	4.08	1.57	-
Leu 29	8.29	4.17	2.06	Hγ 1,61; δCH_3 0.93
Leu 30	8.32	4.14	1.99	Hγ 1.60; δCH_3 0.93
Leu 31	7.67	4.35	1.67; 1.91	δCH_3 0.90
Gly 32	7.93	3.87; 4.04	-	-
Leu 33	7.97	4.50	1.59; 1.70	δCH_3 0.89; 0.94

For methylene and isopropyl groups two chemical shifts are given only when two resonance lines could be identified. ni, not identified.

Supplementary Table 3: Survival and compliance for preterm newborn rabbits treated with either 200 mg kg^{-1} (80 mg mL^{-1}) of Curosurf® or 2% rSP-C33Leu in DPPC:POPG (68:31) and ventilated for 4 hours.

Surfactant	Compliance (mL/(kg x cmH_20)	Survival
Curosurf®	0.48 ± 0.14	12/13
2% rSP-C33Leu	0.40 ± 0.09	12/13

Rabbits (gestational age 29 days, term 31 days) were ventilated for 4 hours with individual pressures to keep tidal volumes between 6 – 7 mg kg^{-1} and survival and compliance at the end of experiments were registered. Compliance is given as mean ± SD.

Supplementary Table 4: *In vitro* surface activity of suspensions (10 mg mL^{-1}) containing Curosurf® or 2% rSP-C33Leu in DPPC:POPG (68:31) measured in a captive bubble surfactometer.

**Sample, n=3 for all preparations**	**Compression needed to reach 5 mN m^{-1} (area, %)**	**γ_{min} (mN m^{-1})**	**γ_{max} (mN m^{-1})**
Curosurf®	16* (9-17)	1.7 (1.1-1.7)	25* (25-29)
2% rSP-C33Leu	26 (21-27)	1.3 (0.8-1.8)	34 (32-38)

Compression is measured as the difference in bubble area between maximum surface tension and when surface tension less or equal to 5 mN m^{-1} is reached. Presented values for minimum and maximum surface tensions are from the fifth cycle of quasi-static dynamics. Values are given as median (range). Significant differences are indicated with one (*) asterisk (t-test, $p < 0.05$).

SUPPLEMENTARY REFERENCES

1. Lukovic, D. et al. Production and characterisation of recombinant forms of human pulmonary surfactant protein C (SP-C): Structure and surface activity. *Biochim. Biophys. Acta* **1758**, 509-518 (2006).
2. Finder, V.H., Vodopivec, I., Nitsch, R.M. & Glockshuber, R. The recombinant amyloid-beta peptide Abeta1-42 aggregates faster and is more neurotoxic than synthetic Abeta1-42. *J. Mol. Biol.* **396**, 9-18 (2010).
3. Garai, K., Crick, S.L., Mustafi, S.M. & Frieden, C. Expression and purification of amyloid-beta peptides from Escherichia coli. *Protein Expr. Purif.* **66**, 107-112 (2009).
4. Long, F., Cho, W. & Ishii, Y. Expression and purification of 15N- and 13C-isotope labeled 40-residue human Alzheimer's beta-amyloid peptide for NMR-based structural analysis. *Protein Expr. Purif.* **79**, 16-24 (2011).
5. Chhetri, G., Pandey, T., Chinta, R., Kumar, A. & Tripathi, T. An improved method for high-level soluble expression and purification of recombinant amyloid-beta peptide for in vitro studies. *Protein Expr. Purif.* **114**, 71-76 (2015).
6. Zhang, L. et al. Expression, purification, and characterization of recombinant human beta-amyloid42 peptide in Escherichia coli. *Protein Expr. Purif.* **64**, 55-62 (2009).
7. Lee, E.K., Hwang, J.H., Shin, D.Y., Kim, D.I. & Yoo, Y.J. Production of recombinant amyloid-beta peptide 42 as an ubiquitin extension. *Protein Expr. Purif.* **40**, 183-189 (2005).
